# Hospitalization and Predictors of Inpatient Mortality among HIV-Infected Patients in Jimma University Specialized Hospital, Jimma, Ethiopia: Prospective Observational Study

**DOI:** 10.1155/2020/1872358

**Published:** 2020-05-27

**Authors:** Kirubel Minsamo Mishore, Nezif Hussein, Solomon Assefa Huluka

**Affiliations:** ^1^School of Pharmacy, College of Health and Medical Sciences, Haramaya University, Harar, Ethiopia; ^2^Department of Pharmacy, College of Public Health and Medical Sciences, Jimma University, Jimma, Ethiopia; ^3^Department of Pharmacology and Clinical Pharmacy, School of Pharmacy, Addis Ababa University, P.O.Box 1176, Addis Ababa, Ethiopia

## Abstract

Despite the number of patients enrolled in ART is increased, HIV/AIDS continues to constitute a significant proportion of medical admissions and risk of mortality in low- and middle-income countries. As one of these countries, the case in Ethiopia is not different. The aim of this study was thus to assess reasons for hospitalization, discharge outcomes, and predictors of inpatient mortality among people living with HIV (PLWH) in Jimma University Specialized Hospital (JUSH), Jimma, Southwest Ethiopia. Prospective observational study was conducted in medical wards of JUSH from February 17^th^ to August 17^th^, 2017. In this study, 101 PLWH admitted during the study period were included. To identify the predictors of mortality, multiple logistic regression analysis was employed. Of the 101 hospitalized PLWH, 62 (61.4%) of them were females and most of them (52.5%) were between 25 and 34 years of age. A majority (79.2%) of the study participants were known HIV patients, before their admission. Tuberculosis (24.8%), infections of the nervous system (18.8%), and pneumonia (9.9%) comprised more than half of the reasons for hospitalization. Moreover, drug-related toxicity was a reason for hospitalization of 6 (5.9%) patients. Outcomes of hospitalization indicated that the overall inpatient mortality was 18 (17.8%). The median CD4 cell counts for survivors and deceased patients were 202 cells/*μ*L (IQR, 121–295 cells/*μ*L) and 70 cells/*μ*L (IQR, 42–100 cells/*μ*L), respectively. Neurologic complications (AOR = 13.97; 95% CI: 2.32–84.17, *P* = 0.004), CD4 count ≤ 100 cells/*μ*l (AOR = 16.40; 95% CI: 2.88–93.42, *P* = 0.002), and short hospital stay (AOR = 12.98, 95% CI: 2.13–78.97, *P* = 0.005) were found to be significant predictors of inpatient mortality. In conclusion, opportunistic infections are the main reason of hospitalization in PLWH.

## 1. Introduction

Globally, close to 35 million people are believed to live with HIV. Sub-Saharan Africa, worst affected region, in particular accounts for 71% of HIV infections [1]. Life expectancy for patients infected with HIV has improved significantly in the era of highly active antiretroviral therapy (HAART) [2]. The decline in hospitalization due to HAART, however, has been unevenly distributed and inconsistent. Despite the global decrease in AIDS-related death and improvement of access to HAART, eastern and southern Africa remain the most HIV-affected regions [3]. In such low- and middle-income countries, HIV and its associated immunosuppression (AIDS) continue to constitute a significant deal of morbidity and mortality in adults. In some of these countries, the problem is acute [4–6].

In resource-poor settings, between 20% and 52% of hospital beds in medical wards are occupied by HIV-infected patients at any given time, mostly with opportunistic infections [7] and ended up with longer hospital stays [8]. Furthermore, it is reported that non-HIV-related hospitalizations of HIV-infected patients is increasing, globally [9]. Most reports of hospitalization from HIV infection in the era of HAART are from the developed countries [10–13]. This is mainly because publications reporting HIV-related hospitalization from developing countries are infrequent.

Data on the spectrum of both HIV- and non-HIV-related illnesses that result in hospital admission are essential for policymakers and stakeholders to plan actions in reducing morbidity, mortality, and further hospitalization [14]. Recently, Negera and Mega [15] reported that body mass index (BMI) of less than 18.5 is a significant predictor of inpatient mortality in Ethiopia. However, with the paucity of published data on HIV/AIDS in Ethiopia, little is known about other reasons for hospitalizations, discharge outcomes, and predictors of inpatient mortality in hospitalized patients with HIV. Thus, this study aimed to assess reasons for hospitalization, discharge outcomes, and predictors of inpatient mortality among people living with HIV in Jimma University Specialized Hospital (JUSH), Jimma, Southwest Ethiopia.

## 2. Methods

### 2.1. Study Area and Period

This study was conducted from 17^th^ February to 14^th^ August 2017 in medical wards of JUSH, which is the only teaching and referral hospital in Southwest Ethiopia. The hospital provides services for approximately 9000 inpatient and 80,000 outpatient attendants a year from the catchment population of about 15 million people. It has more than 450 beds. In this hospital, the HIV test was performed using the HIV 1/2 STAT-PAK1 RDT (Chembio Diagnostics, Medford, NY, USA) kit. In JUSH, *Mycobacterium tuberculosis* diagnosis was made using the Xpert assay (Cepheid Xpert MTB/RIF®).

### 2.2. Study Design and Participants

A hospital-based prospective observational study was employed. The study population included 101 patients who met the following criteria: HIV seropositive (either known prior to hospitalization or tested positive following hospitalization), adult patients (≥15 years), admitted to medical wards of JUSH in the study period, willing to participate in the study, and stayed for at least 24 hours in the inpatient wards. To confirm HIV status of patients, every hospitalized patient underwent provider-initiated counseling and testing (PICT).

### 2.3. Data Collection and Data Quality

Patient demographics, anthropometric measurements, reason for hospitalization, comorbidities, complications, laboratory profile, and HAART status were collected using a predesigned data collection form. All CD4 cell counts included in study analyses were either done during hospitalization or within the previous 1 month before hospitalization. The clinical staging of patients was carried out using WHO guidelines for the clinical staging of HIV/AIDS for adults.

Adherence to HAART was assessed for 46 patients who were on HAART for at least 6 months prior to their admission. Adherence was estimated from patients' self-report of missed doses out of 30 doses of their prescribed medication and reported as good (≥95%), fair (85 to 95%), or poor (<85%) if and only if they missed 2 and less, 3 to 5, or 6 and more doses, respectively [16]. Data were collected by hospital pharmacists, working in medical wards, after being trained on interview techniques, data collection methods, and techniques of measurements. Moreover, to determine the outcomes of hospitalization, they followed the patients prospectively until discharged or died.

### 2.4. Data Processing and Analysis

Data were coded, entered, cleaned, and analyzed using SPSS version 20 statistical package. Bivariate and multivariate logistic regression analyses with 95% confidence interval were employed in order to infer associations and predictions. In bivariate analysis, all explanatory variables that are associated with the outcome variable (inpatient mortality) with a *P* value of <0.2 were included in the final logistic model. *P* value < 0.05 was considered as statistically significant for all the independent variables in the final model.

### 2.5. Ethical Consideration

Letter of ethical clearance was obtained from the Ethical Review Board of College of Public Health and Medical Sciences, Jimma University. Informed, voluntary, written, and signed consent/assent was obtained from each study participants/caregivers. Privacy and confidentiality were strictly maintained throughout the study.

## 3. Results

### 3.1. Sociodemographic Characteristics of the Study Population

Of 101 hospitalized PLWH enrolled in the study, 62 (61.4%) of them were females and 53 (52.5%) of the patients were between 25 and 34 years of age. Most of the study participants were urban residents (64.4%) and unemployed (62.4%). As it is illustrated in Table 1, the measured mean BMI of patients was 17.63±3.24 kg/m^2^. Slightly more than one-third of the patients (34.8%) had severe malnutrition (BMI < 16 kg/m^2^).

### 3.2. Clinical Characteristics of the Participants

Clinical characteristics and laboratory findings of the patients are presented in Tables 2 and 3, respectively. The result showed that 21 (20.8%) of participants were newly diagnosed HIV-positive patients (tested on hospitalization). For known HIV patients (79.2%), the median duration of time since their diagnosis was 24 months (IQR, 6–60). Majority (82.2%) of the patients were in WHO clinical stage 4 and 44.6% of them had complications. The main (46.7%) complication of the hospitalized patient was severe neurologic dysfunctions. More than a quarter (27.7%) of the participants had a history of prior hospitalization in the last 12 months, and opportunistic infections were the leading (53.6%) reasons for their previous hospitalization (Table 2).

In this study, 29 (28.7%) of the patients had CD4 cell counts of ≤100 cells/*μ*L (IQR, 93.5–279.0). The median CD4 cell count for survivors and died was 202 cells/*μ*L (IQR, 121–295) and 70 cells/*μ*L (IQR, 42–100), respectively (Table 3). Anemia was reported in 88 of 96 (85.4%) patients (defined as hemoglobin (Hg) < 13 gm/dL for males and <12 gm/dL for females), and it was severe (Hg < 8gm/dL) in 21 (21.9%) of them.

### 3.3. Antiretroviral Therapy Regimen of Patient on HAART

Among 80 known HIV patients, 65 (81.2%) of them were on HAART with the median duration of 19.00 (IQR, 3.25–40.25) months and majority (89.2%) of them were on first-line regimen. About a third (16; 34.8%) of the patients,who were assessed for adherence, had a poor (<85%) HAART adherence. Among 19 (29.2%) patients who had regimen changes, treatment failure was found to be the leading (36.8%) reason for treatment switch (Table 4).

### 3.4. Reasons for Hospitalization and Treatment Outcome of Hospitalized Patients

**Figure 1:**
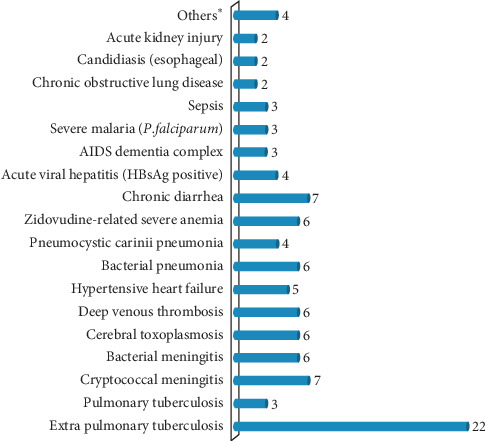
Reasons for hospitalization of PLWH to medical wards of JUSH, Jimma, Southwest Ethiopia, 2017 (*N* = 101). ∗Chronic liver disease, herpes zoster, cellulitis, and disseminated Kaposi sarcoma each accounts for 1.

**Figure 2:**
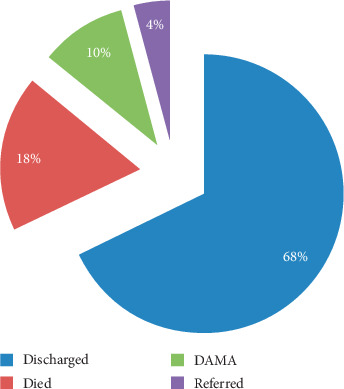
Discharge outcomes of PLWH admitted to the medical wards of JUSH, Jimma, Southwest Ethiopia, 2017 (*N* = 101).

Tuberculosis (TB) was the most common diagnosis that accounted for 25 (24.8%) of the reasons for hospitalization (Figure 1). The median duration of hospital stay for the patients was found to be 13 days (IQR, 8–20 days). As it is revealed in Figure 2, 82.2% of the admitted patients survived: 69 (68.3%) discharged, 10 (9.9%) of them discharge against medical advice (DAMA), and 4 (4.0%) transferred cases. The remaining 18 (17.8%) were deceased, of whom 12 (66.7%) died within the first 7 days of their hospital stay.

### 3.5. Factors Associated with Inpatient Mortality

Both univariate and multivariate logistic regression analyses (Table 5) showed that presence of neurologic complications, CD4 count ≤ 100 cell/*μ*L, and hospital stay of less than 7 days were predictors of inpatient mortality. PLWH hospitalized with neurologic complications were almost fourteen times more likely to die inpatient compared with those who were not (AOR = 13.97; 95% CI: 2.32–84.17, *P*=0.004). The odds of dying inpatient were significantly (*P*=0.002) higher in PLWH hospitalized with CD4 count ≤ 100 cells/*μ*l compared with CD4 count > 100 cells/*μ*L (AOR = 16.40; 95% CI: 2.88–93.42).

## 4. Discussion

In this study, opportunistic and other infectious diseases were dominant attributes of hospitalization. The spectrum of opportunistic infection is in agreement with previous reports on hospitalized HIV/AIDS patients from other parts of Ethiopia [17–19] and other low- and middle-income countries [3, 5, 20–23]. Our study, however, reported a relatively lower proportion of TB. This could be because of the better availability of free HAART, which determines the frequency and severity of opportunistic infections such as active TB disease [7, 18, 24].

The prevalence of CNS infections observed during our study period was 18.8%. This is in line with a similar study conducted in Kenya [25] and lower than other studies [26, 27]. The common CNS infections identified were cryptococcal meningitis (36.8%), bacterial meningitis (31.6%), and cerebral toxoplasmosis (31.6%). This proportion of CNS infections was consistent with other studies [3, 15, 20, 28–30]. HAART-related toxicity was also among the commonly occurred reasons for hospitalization. Proper counseling about the adverse effects of antiretroviral drugs and aggressive monitoring of patients before and within the first few weeks of commencement of HAART will help to reduce morbidity associated with the use of these drugs.

The overall inpatient mortality in our study population was 17.8%, similar to previous reports [3, 23, 31]. Our finding, nevertheless, was higher than previous studies in India [6] and France [30]. The high mortality is probably reflective of the advanced nature of the disease during hospitalization [10, 20]. Although there were differences in study design, a higher mortality rate was reported in other studies [16, 20, 21, 32].

Logistic regression analysis showed that presentation with neurologic complications, low CD4 count (≤100 cells/*μ*l), and short duration of hospital stay (<7 days) were predictors of inpatient mortality. Multiple studies reported a statistically significant association of low CD4 cell counts as the predictor of inpatient mortality [20, 33]. HIV patients hospitalized with neurologic complications were almost 14 times more likely to die inpatient compared with those without neurologic complications. This is in accordance with findings from other studies from Ethiopia by Berhe et al. [34] and elsewhere by Gill et al. [35].

Our study has some limitations that should be considered while interpreting the findings. They include the following: certain disease conditions might have been overestimated or underestimated due to inadequate diagnostic facilities. Outcome measures for our study depended on the survival status at last contact with our patient in the hospital; we cannot exclude an underrepresentation of mortality rate as some DAMA patients might have died outside our hospital.

## 5. Conclusion

Tuberculosis, infections of the nervous system, and pneumonia were the top three leading reasons for hospitalization. Furthermore, our study disclosed that presentation with neurologic complication, low CD4 count, and short hospital stay were found to be predictors of inpatient mortality.

## Figures and Tables

**Table 1:** Sociodemographic characteristics of PLWH hospitalized to the medical wards of JUSH, Jimma, Southwest Ethiopia, 2017 (*N* = 101).

Characteristics	Frequency	Percent
Sex		
Male	39	38.6
Female	62	61.4

Age group		
15–24	8	7.9
25–34	53	52.5
35–44	25	24.7
45+	15	14.9

Residence		
Urban	65	64.4
Rural	36	35.6

Level of education		
No education	29	28.7
Primary	42	41.6
Secondary+	30	29.7

Marital status		
Single	15	14.8
Married	50	49.5
Divorced	25	24.8
Widowed	11	10.9

Occupation		
Government employee	10	9.9
Self-employed	28	27.7
Unemployed	63	62.4

Body mass index (in kg/m^2^)		
<16	35	34.7
16–18.5	23	22.7
18.5–24.9	43	42.6
≥25	0	0.0

**Table 2:** Clinical characteristics of PLWH hospitalized to medical wards of JUSH, Jimma, Southwest Ethiopia, 2017.

Characteristics	Category	Frequency	Percent
HIV status at admission	Known	80	79.2
	New	21	20.8

Duration of HIV in month (*N* = 80)	<6 months	21	26.3
	≥6 months	59	73.7

Clinical stage of HIV/AIDS on hospitalization	Stage 1	5	5.0
	Stage 2	2	2.0
	Stage 3	13	13.0
	Stage 4	81	80.2

Complication (*N* = 45)	Neurologic	21	46.7
	Respiratory	14	31.1
	Hypovolemic shock	2	4.4
	Hepatic encephalopathy	2	4.4
	Gastric bleeding	2	4.4
	Nephropathy	2	4.4

Comorbidity (*N* = 22)	Cardiovascular	8	36.4
	Gastrointestinal	5	22.7
	Urologic	5	22.7
	Respiratory	4	18.2

Reasons for hospitalization (known HIV-positive patients) in the last 12 months (*N* = 28)	Opportunistic infections	15	53.6
	DVT	2	7.1
	COPD∗	2	7.1
	Malaria	2	7.1
	Bacterial meningitis	2	7.1
	Not specified	5	17.9

^*∗*^Chronic obstructive pulmonary disease; DVT = deep venous thrombosis.

**Table 3:** Laboratory profile of PLWH hospitalized to the medical wards of JUSH, Jimma, Southwest Ethiopia, 2017.

Parameters	Median (IQR)	References
Hemoglobin (gm/dL) (*N* = 96)	9.75 (8.50–11.50)	12.0–17.0
Hematocrit (%) (*N* = 96)	30.24 (26.19–34.70)	40.0–54.0
Platelet (×10^9^/L) (*N* = 93)	252.0 (149.50–402.0)	150–500
Aspartate aminotransferase (unit/L) (*N* = 77)	40.0 (21.40–80.50)	0–38
Alanine aminotransferase (unit/L) (*N* = 76)	25.25 (16.05–45.83)	0–40
Serum creatinine (mg/dL) (*N* = 70)	0.85 (0.68–1.29)	0.8–1.2
Blood urea nitrogen (mg/dL) (*N* = 70)	24.13 (14.38–41.54)	8–20
CD4 count (cells/*μ*L) (*N* = 101)	193.0 (93.50–279.0)	500–1,500

**Table 4:** Antiretroviral therapy related characteristics of PLWH admitted to medical wards of JUSH, Jimma, Southwest Ethiopia, 2017.

Characteristics	Category	Frequency	Percent
Prior HAART use (*N* = 80)	Yes	65	81.2
	No	15	18.8

Type of HAART regimen (*N* = 65)	First line	58	89.2
	Second line	7	10.8

First-line regimen (*N* = 58)	TDF + 3TC + EFV	37	63.8
	AZT + 3TC + NVP	11	19.0
	TDF + 3TC + NVP	5	8.6
	Others	5	8.6

Second-line regimen (*N* = 7)	ABC + ddi + LPV/r	6	85.7
	ABC + 3TC + LPV/r	1	14.3

Adherence status among HAART users for ≥6 months (*N* = 46)	Good	28	60.9
	Fair	2	4.3
	Poor	16	34.8
History of regimen change (*N* = 65)	Yes	19	29.2
	No	46	70.8

Reason for regimen change(*N* = 19)	Treatment failure	7	36.8
	Toxicity/side effects	6	31.6
	Due to new TB	3	15.8
	Others	3	15.8

Prior co-trimoxazole prophylaxis in known HIV patients (*N* = 80)	Yes	51	63.8
	No	29	36.2

**Table 5:** Univariate and multivariate analyses of factors associated with inpatient mortality among PLWH admitted to the medical wards of JUSH, Jimma, Southwest Ethiopia, 2017 (*N* = 101).

Variables	Category	Died (*N* = 18)	Survived (*N* = 83)	COR (95% CI)	*P* value∗	AOR (95% CI)	*P* value^*∗*^
Sex	Male	10 (25.6%)	29 (74.4%)	2.33 (0.83–6.54)	0.109	0.57 (0.09–3.43)	0.539
	Female	8 (12.9%)	54 (87.1%)	1.000		1.000	

Neurologic complication	Yes	13 (61.9%)	8 (38.1%)	24.38 (6.89–86.19)	0.000	13.97 (2.32–84.17)	0.004
	No	5 (6.2%)	75 (93.8%)	1.000	0.000	1.000	

CD4 count	≤100	14 (48.3%)	15 (51.7%)	15.9 (4.57–6)	0.001	16.40 (2.88–93.42)	0.002
	>100	4 (5.6%)	68 (94.4%)	1.000		1.000	

Hospital stay in days	<7	12 (46.2%)	14 (53.8%)	9.86 (3.17–30.69)	0.000	12.98 (2.13–78.97)	0.005
	≥7	6 (8.0%)	69 (92.0%)	1.000		1.000	

COR = crude odds ratio; AOR : adjusted odds ratio; CI: confidence interval; ^*∗*^*P* value <0.05 indicates a statistically significant association.

## Data Availability

The data used to support the finding of this study are available from the corresponding author upon request.

## Abbreviations

AIDS: Acquired immunodeficiency syndrome
CNS: Central nervous system
COPD: Chronic obstructive pulmonary disease
DAMA: Discharge against medical advice
DVT: Deep venous thrombosis
HAART: Highly active antiretroviral therapy
HIV: Human immunodeficiency virus
IQR: Interquartile range
JUSH: Jimma University Specialized Hospital
PLWH: People living with HIV
TB: Tuberculosis.
